# The Concussion, Exercise, and Brain Networks (ConExNet) study: a cohort study aimed at understanding the effects of sub-maximal aerobic exercise on resting state functional brain activity in pediatric concussion

**DOI:** 10.1186/s13102-024-00926-1

**Published:** 2024-06-17

**Authors:** Bhanu Sharma, Eric Koelink, Carol DeMatteo, Michael D. Noseworthy, Brian W. Timmons

**Affiliations:** 1 Child Health and Exercise Medicine Program, Department of Pediatrics, McMaster University, Hamilton, ON Canada; 2 School of Rehabilitation Science, McMaster University, Hamilton, ON Canada; 3 CanChild Centre for Childhood Disability Research, McMaster University, Hamilton, ON Canada; 4 Imaging Research Centre, St. Joseph’s Healthcare, Hamilton, ON Canada; 5 Department of Electrical & Computer Engineering, McMaster University, Hamilton, ON Canada; 6 McMaster School of Biomedical Engineering, McMaster University, Hamilton, ON Canada; 7 Department of Medical Imaging, McMaster University, Hamilton, ON Canada; 8 Department of Pediatric Emergency Medicine, McMaster Children’s Hospital, Hamilton, ON Canada

**Keywords:** Concussion, Brain injury, Pediatric, Children, MRI, Functional MRI, Exercise, Exercise medicine

## Abstract

**Background:**

Recent scientific evidence has challenged the traditional “rest-is-best” approach for concussion management. It is now thought that “exercise-is-medicine” for concussion, owing to dozens of studies which demonstrate that sub-maximal, graded aerobic exercise can reduce symptom burden and time to symptom resolution. However, the primary *neuropathology* of concussion is altered functional brain activity. To date, no studies have examined the effects of sub-maximal aerobic exercise on resting state functional brain activity in pediatric concussion. In addition, although exercise is now more widely prescribed following concussion, its cardiopulmonary response is not yet well understood in this population. Our study has two main goals. The first is to understand whether there are exercise-induced resting state functional brain activity differences in children with concussion vs. healthy controls. The second is to profile the physiological response to exercise and understand whether it differs between groups.

**Methods:**

We will perform a single-center, controlled, prospective cohort study of pediatric concussion at a large, urban children’s hospital and academic center. Children with sport-related concussion (aged 12–17 years) will be recruited within 4-weeks of injury by our clinical study team members. Key inclusion criteria include: medical clearance to exercise, no prior concussion or neurological history, and no implants that would preclude MRI. Age- and sex-matched healthy controls will be required to meet the same inclusion criteria and will be recruited through the community. The study will be performed over two visits separated by 24–48 h. Visit 1 involves exercise testing (following the current clinical standard for concussion) and breath-by-breath gas collection using a metabolic cart. Visit 2 involves two functional MRI (fMRI) scans interspersed by 10-minutes of treadmill walking at an intensity calibrated to Visit 1 findings. To address sub-objectives, all participants will be asked to self-report symptoms daily and wear a waist-worn tri-axial accelerometer for 28-days after Visit 2.

**Discussion:**

Our study will advance the growing exercise-concussion field by helping us understand whether exercise impacts outcomes beyond symptoms in pediatric concussion. We will also be able to profile the cardiopulmonary response to exercise, which may allow for further understanding (and eventual optimization) of exercise in concussion management.

**Trial registration:**

Not applicable.

**Supplementary Information:**

The online version contains supplementary material available at 10.1186/s13102-024-00926-1.

## Introduction

Concussions are a form of traumatic brain injury (TBI) that pose considerable healthcare burden [1, 2]. Recent incidence estimates, based on physician billing data from Ontario, Canada, suggest an annual incidence of 1,153/100,000 residents [3]. This injury impacts individuals throughout the lifespan, though the incidence of concussion is amongst the highest in pediatric populations [3, 4], a group in which symptoms often also take longer to resolve [5, 6]. In pediatric concussion, greater symptom severity is a predictor of protracted recovery (taking 30-days or longer) [6, 7], and the injury is also often co-morbid with poorer mental health [8] and quality of life [9–11]. Despite all this, treatment options for concussion have historically remained limited [12–14].

Until recently, concussion management was governed by a “rest-is-best” approach, which is now being supplanted by an “exercise-is-medicine” mindset [15]. More specifically, graded, sub-maximal aerobic exercise (performed on a stationary bike or treadmill) is becoming part of best clinical practice [16], with dozens of studies now supporting the use of exercise to reduce concussion symptoms and/or time to recovery. A recent meta-analysis of 23 studies showed that sub-maximal aerobic exercise had a large effect (*Hedge’s g* = 1.71) on concussion recovery, operationalized as a reduction in concussion symptoms [17]. Adding to this, another recent systematic review and meta-analysis of randomized controlled trials in the field (*n* = 7; *N* = 326) reached a similar conclusion, finding that sub-maximal aerobic exercise significantly improved symptoms (standard mean difference [SMD]: -0.44 [-0.68, -0.19]) [18]. There is a substantial and growing evidence base that supports the use of sub-maximal aerobic exercise as a treatment option for concussion symptoms. Yet the field recognizes that symptom recovery does not equate to physiological recovery from concussion [19].

Concussions, by definition, are not associated with structural injury; rather, their primary neuropathology is functional neurological disturbance [16]. As such, numerous studies have reported that there are functional neuropathological disturbances (typically observed using resting-state functional MRI; rsfMRI) in pediatric concussion [20–25]. Recently, the largest neuroimaging cohort of pediatric concussion reported that in comparison to children with orthopedic injury (who served as an injury-control group), children with concussion had regional and global network abnormalities in the rsfMRI signal (as computed using graph theory metrics including degree centrality, efficiency, and clustering coefficient), which persisted in some cases up to 6-months post-injury [26]. Functional neurological disturbance is commonplace in concussion, even in those who are asymptomatic [19]. To date, no studies have examined the effects of exercise on functional brain activity in pediatric concussion. Analogous studies, however, have been performed in adults. More specifically, two studies (on one cohort of adults with mild TBI) found that an aerobic exercise stimulus (similar to that being currently prescribed for concussion management) led to hypo-connectivity between several brain regions, including those that comprise the default mode network [27, 28]. Similar data are unavailable in pediatric concussion, leaving a gap in our understanding of how exercise impacts the concussed pediatric brain. Whether such functional neurological disturbance translates to sport-specific consequences, such as gait abnormalities as measured by accelerometers, also requires further study in pediatric concussion. Further, despite increased interest in prescribing exercise as a treatment for concussion, the role of exercise testing as a diagnostic or prognostic tool has remained relatively understudied, leaving another important knowledge gap. To date, two studies have collected exercise physiology data while children with concussion performed sub-maximal aerobic exercise [29, 30]. The first of these reported no groupwise differences (i.e., concussion vs. healthy) in resting heart rate, systolic and diastolic blood pressure, or changes in V̇O_2_, V̇CO_2_, or minute ventilation from baseline to test termination; only ratings of perceived exertion significantly differed (29). Similarly, the second study reported no groupwise differences in the outcomes they collected, namely: heart rate change throughout the test, blood pressure responses to exercise, or differences in end-tidal O_2_ or CO_2_ [30]. Additional research is required to add to these findings and provide initial insights into how sub-maximal metrics (such as the oxygen uptake efficiency slope; OUES [31] respond to exercise in pediatric concussion. Further, while there are data on gait and balance in concussion [32, 33], studies have not yet examined gait differences between children with concussion and healthy controls during a controlled, progressive sub-maximal aerobic exercise session. Such accelerometer data, combined with exercise physiology data, may offer additional insight into how gait varies as a function of exercise intensity of perceived exertion, which can have implications for return-to-sport.

To address these knowledge gaps, we performed a prospective, single-center controlled cohort study involving children with a sport-related concussion (aged 12–17) and age- and sex-matched healthy peers. The primary objective of our study was to understand the effects of a single bout of aerobic exercise (modelled on the current standard for exercise testing in concussion [16]) on rsfMRI network connectivity. We hypothesized, based on analogous data in adults, that exercise would lead to generalized patterns of hypo-connectivity in those with a concussion. Our second objective was to use breath-by-breath exercise physiology (collected during a progressive, sub-maximal aerobic treadmill test) to understand cardiorespiratory response to graded aerobic exercise in this population. Per prior research, we hypothesized that children with concussion would perceive exercise to be more difficult in comparison to their peers. Further, given the known autonomic dysfunction that occurs in concussion [34], we hypothesized that there would be an attenuated cardiopulmonary response to exercise in children with concussion in comparison to healthy controls. A third objective was to understand if there were gait abnormalities in children with concussion (measured using a tri-axial, waist-worn accelerometer) over the duration of a controlled, graded treadmill test. We hypothesized that there would be more variability in the anterior-posterior and medio-lateral planes in children with concussion vs. healthy peers, and that this variation would increase with exercise intensity.

## Methods

### Design

This proposed study is a single-center, prospective cohort study, with fMRI scans immediately before and after a single bout of aerobic exercise. Gait will also be assessed during this bout of exercise using a waist-worn, tri-axial accelerometer. This study will also include a 28-day follow-up period (to monitor daily symptoms and habitual physical activity) beginning after the MRI, as well a healthy control group matched for sex, biological age (to control for the potential confounding effects of maturational status) and sport participation.

### Study participants

*Patients*: *Inclusion criteria*: **1**) Emergency department or urgent care clinic diagnosis of concussion, made by a physician with experience in pediatric brain injury and/or sports medicine in accordance with the diagnostic criteria from recent consensus statements on concussion in sport [16, 35]. **2**) Participants must be within four-weeks of injury at the time of recruitment. **3**) Age of 12 to 17 years. Our group has found that children younger than 12 have difficulty laying still during the MRI, resulting in motion artifacts that may limit data quality. The age limit for pediatric clinics at our children’s hospital is 17. While there are neurodevelopmental changes occurring throughout this age group, children of this age range are expected to be in a similar neurodevelopmental window; biological age will nonetheless be used as a moderator variable in analyses. **4**) Medical clearance (per the emergency department physician) to perform aerobic exercise test on a treadmill. **5**) Fluency in English to provide informed assent and consent (for parents) and ensure symptom report validity (as measures are validated in English). *Exclusion criteria*: **1**) Previous history of suspected concussion or other neurological comorbidity (as per self-report) that may confound neuroimaging findings. **2**) Complicated mild or moderate-to-severe brain injuries. **3**) Mobility, gait, or vestibular impairments that preclude safe exercise. **4**) Claustrophobia or post-surgical metallic inclusions (e.g., pacemaker, neurostimulators, aneurism clips) that may prevent safe MRI testing.

*Healthy controls*: *Inclusion criteria*: **1**) Age of 12 to 17 years. **2**) Fluency in English to provide informed consent and ensure symptom report validity. *Exclusion criteria*: **1**) Present or previous history of concussion or other neurological comorbidity (as per self-report). **2**) Mobility, gait, or vestibular impairments that preclude safe exercise. **3**) Claustrophobia or metal implants/devices that are contraindicated for MRI testing.

### Recruitment strategy

#### Patients

Children with concussion will be recruited from the McMaster Children’s Hospital Emergency Department and an associated Urgent Care Clinic. We selected these centers as our recruitment sites because: **1**) they have a large catchment area, with an annual throughput of approximately 250 children with concussion (or 5 patients per week) within the age range of interest, **2**) it permits recruitment while participants are in the acute stages of concussion, **3**) our already strong and positive relationship with emergency medicine physicians (who are experienced in pediatric concussion) will facilitate recruitment, **4**) they are based in a metropolitan area with a population of ~ 750,000, and our sample is thus estimated to draw from a representative population which precludes the need for stratified sampling.

#### Healthy controls

Healthy controls will be recruited through advertisement in community settings (e.g., local sports facilities, youth clubs) and through our existing networks, including mailing lists of individuals who agreed to be contacted about participation in future research studies.

### Study protocol

Data will be collected over 30-days, including 2 study visits (separated by 24-48-hours to ensure physiological recovery following exercise) and a 28-day follow-up period for symptom monitoring. The purpose of Visit 1 is to assess exercise tolerance of each participant so that the intensity of the exercise test to be performed on Visit 2 can be calibrated to individual capacity. The purpose of Visit 2 is to assess the effects of exercise on resting state functional brain networks.

#### Visit 1

The first study visit will take place in the exercise laboratory of the Child Health and Exercise Medicine Program at McMaster Children’s Hospital. Participants and their parents will first complete assent and consent forms, as well as demographic and medical intake questionnaires. Participants will then perform a baseline exercise test of aerobic fitness (the Buffalo Concussion Treadmill Test; BCTT), detailed below.

#### Visit 2

The second study visit will occur at the Imaging Research Centre at St. Joseph’s Healthcare, Hamilton. Here, participants will first complete a baseline MRI (as per the imaging parameters in the *Measurements* section). Participants will then exercise on a treadmill adjacent to the MRI room for 10 min at an intensity equivalent to 85% of the maximum intensity achieved during the exercise test performed at Visit 1. Participants will then re-enter the MRI (which can be done in under 150 s, as per our pilot data) for post-exercise scanning using the same fMRI protocol. Physiological metrics will be continuously monitored and recorded throughout imaging using MRI compatible pulse oximeter. Based on our pilot data (including 5 children with concussion and 4 healthy controls), we have not encountered any difficulties with the MRI (with respect to excessive motion or in-and-out times) and all participants tolerated the duration of our imaging protocol. Each participant will be given a waist-worn accelerometer to measure habitual physical activity, along with wear instructions for the next 28-days.

#### Follow-up

Participants will continue to wear the accelerometer for 28-days after Visit 2; they will then return the accelerometers to our lab in a pre-paid return envelope. During these 28 days, participants will also self-report concussion symptoms using an online version of the PCSS [36] using a secure survey system (RedCAP) every 24 h.

### Measurements

*Aerobic fitness*: This exercise test will follow the parameters of the BCTT, the current standard for evaluating exercise tolerance in concussion. This progressive test involves an increase in incline of 1%/min and a starting speed of either 3.2 or 3.6 mph. The test is terminated when participants self-report maximum exertion, or report a rating of perceived exertion (RPE) ≥ 17 per the Borg RPE Scale [37]. The BCTT is also terminated if patients report a ≥ 3-point increase in concussion symptoms (relative to baseline) on a 10-point visual scale, which is an abbreviated and adapted version of the PCSS. The test is also terminated if the participant reports any distress or discomfort (irrespective of RPE or symptom score). RPE and symptom scores will be recorded every 2-minutes throughout the test. The BCTT has high test-retest reliability (ICC = 0.79) as well as high sensitivity (99%) and specificity (89%) for identifying and ruling out concussion symptoms, respectively [38].

While the BCTT has been widely used to assess aerobic capacity (based on heart rate) in concussion, to the best of our knowledge ours will be one of the first studies to measure inspired/expired O_2_ and CO_2_ continuously throughout the BCTT using a calibrated metabolic cart (Vmax29, SensorMedics, Yorba Linda, CA, U.S.A.). This permits the first opportunity for a breath-by-breath gas analysis during the BCTT, and thus objective measurement of typical exercise testing parameters (e.g., ventilatory threshold, oxygen uptake efficiency slope). Participants will continue to exercise until they reach a symptom-limited threshold. Heart rate will be monitored continually throughout the test and during a 2-minute post-exercise recovery period.

*Anatomical and functional MRI*: Imaging will be performed using a GE 3 Tesla MR750 Discovery MRI scanner and 32-channel phased array RF coil (General Electric Health Care, Milwaukee, WI). Anatomical images will be collected using a 3D IR-prepped fast SPGR T1-weighted sequence (TR/TE = 7.5/2.1ms, TI = 450ms, flip angle = 12°, 512 × 512 matrix, 140–160 slices, 24 cm FOV). Resting state fMRI data (axial 2D acquisition, gradient echo EPI, TR/TE = 2000/35ms, flip angle = 90°, 64 × 64 matrix, 31–35 slices, 300 time points (10 min), 24 cm FOV), will be acquired wherein participants will be asked to remain awake, keep their eyes open, and not to think of anything in particular. A B_o_ map will be acquired for resting state scans, using the same geometric prescription. With regards to logistics, subjects the 3D, resting state and B_o_ maps will be acquired prior to exercise. Immediately following exercise (outside of the scanner) resting state, its corresponding B_o_ map and lastly a 3D scan will be acquired. Preprocessing will be performed using CONN 21a [39], which runs on the functionality of SPM12 [40] and MATLAB [41]. Data from each participant will be corrected for B_0_ field inhomogeneities. All functional data will be registered to the participant’s high resolution T1-weighted anatomical scans, then subsequently registered to MNI152 standard space at 2 mm resampling resolution. BOLD images will be high-pass filtered (cut off at 0.01 Hz for low level noise removal), and spatially smoothed with a Gaussian kernel with full width at half maximum (FWHM) of 6 mm (to improve signal-to-noise ratio).

*Concussion symptoms (PCSS)*: The PCSS is a 22-item questionnaire [36], wherein patients rank the severity of their somatic, cognitive, emotional, and sleep-related symptoms. The PCSS has high internal consistency (Crohnbach α = 0.87), moderate-high test-retest reliability (*r* = 0.65), high sensitivity and specificity (0.819 and 0.849, respectively), and high face and content validity [42]. Further, the PCSS has an abbreviated version that has been used to assess changes in symptoms during the BCTT [43, 44].

#### Accelerometry

Waist-worn accelerometers will be used to measure gait in 3-axis during the Visit 1 treadmill test. More specifically, prior to starting the test, participants will be fitted with the accelerometer on their right hip by research staff. The accelerometer tracks the entirety of the treadmill test, beginning from warm-up. The planned accelerometer we will use is the ActiGraph GT3x, a small and light-weight device that measures orthogonal vectors of acceleration along x-, y-, and z-axes, at 100 Hz sampling. Of note, this accelerometer measures physical activity in acquired brain injury with high reproducibility [45]. Participants will also be asked to wear the accelerometer for 4-weeks after Visit 2, allowing us to understand their patterns of habitual physical activity. During this follow-up period, participants also will be asked to record via a log book when the device was put on and taken off (e.g., when waking and sleeping, or taking a shower), in order to account for true wear-time.

### Data analyses

Data analysis approaches are summarized by the three main objectives:

1. *Understanding pre-post exercise rsfMRI changes in children with concussion vs. healthy controls.*

The primary analyses will be performed using a Generalized Linear Model (GLM) approach within CONN, with two conditions (pre-exercise and post-exercise) and two groups (concussion vs. healthy). The primary analysis aim is to determine group-wise differences in pre-post fMRI in “core” resting state networks, including the default mode network, salience network, sensorimotor network, and frontoparietal network. As a separate analysis, we will perform a dynamic independent component analysis (dyn-ICA), by means of a sliding-window approach to characterize sources of temporal variability in the BOLD signal. This contrasts traditional ICA approaches that are spatially oriented. The dyn-ICA analyses is ideally suited to our data, as it permits insight into whether there is increased temporal variability in the control group as the post-exercise fMRI progresses, in comparison to controls.

2. *Understanding differences in the cardiopulmonary response to exercise in children with concussion vs. healthy controls.*

The concussion and healthy control group will be compared with respect to a number of the cardiopulmonary metrics listed above. As the exercise test is clinical and terminated on the basis of symptom exertion, analyses will be adjusted for test time (by using the shortest test time duration only, or including all data as a percentage of total test time). Groupwise-differences will be assessed using ANCOVAs (Group x Time), controlling for sex and age. These differences may also be assessed using generalized linear models, to understand the effects of time (or test intensity), age, and sex on cardiopulmonary response to exercise.

3. *Understanding differences in gait during sub-maximal aerobic exercise in children with concussion vs. healthy controls.*

Data from the tri-axial accelerometer will first be cleaned to include only data specific to the treadmill test, from warm-up to test termination. Analyses will focus on understanding whether there is more medio-lateral or anterior-posterior gait variation (operationalized, for example, as the coefficient of variation) in gait in the concussion group, compared to healthy controls. The analysis will aim to determine if there is more gait variation as test intensity (which in this case is represented by treadmill gradient) increases. As above, groupwise differences may be assessed using ANCOVAs (Group x Time), controlling for age and sex, or generalized linear models predicting gait variability on the basis of test time (i.e., intensity), age, and sex.

### Sample size requirements

Sample size calculation for fMRI studies depart from traditional methods, partly because of the number of parameters that can vary voxel-to-voxel, leading to tens of thousands of comparisons made during analysis. Instead, researchers have developed tools for closed form power calculations in fMRI studies which are based on **(i)** the proportion of the brain activated and **(ii)** the average effect size in these activated areas. One such tool is available online at www.neuropowertools.org; the theoretical and mathematical approach this tool uses for fMRI sample size calculations is outlined in an associated publication. Using our pilot data, we generated a statistical map of pre- to post-exercise changes in children with concussion (*n* = 5). This map was uploaded to www.neuropowertools.org and the following parameters were specified, as required: t-statistic screening threshold (2.776), number of participants [5], alpha-level (0.05), and voxel size and smoothness (3.75 × 3.75 × 4.00 mm voxels with Gaussian smoothing at 5 mm FWHM). Using this approach, we will be able to achieve 80% power (using Random Field Theory to correct for multiple comparisons) with a sample of 36 participants per group. To account for the potential of missing data, we will recruit 40 children with concussion and 40 healthy controls.

## Discussion

Our controlled, prospective cohort study is well-positioned to add to the burgeoning field at the intersection of exercise and pediatric concussion. More specifically, the goal of our study is to advance the field providing the first insight into how graded sub-maximal aerobic exercise, similar to that currently being prescribed, impacts resting state brain activity. This will complement the now dozens of studies (ranging from case-series to prospective cohorts to randomized trials) that have demonstrated that sub-maximal aerobic exercise can reduce symptom burden and time to symptom recovery [17, 18, 46]. We will be able to add to this evidence base by examining the effects of exercise on an outcome that is more directly related to the primary neuropathology of concussion, namely functional brain activity [16].

Sub-maximal aerobic exercise is becoming part of clinical management of concussion, with recent international consensus statements advocating for its use after the first 24–48 h of concussion [16]. As such, the field stands to benefit from an understanding of its impact on more than just symptoms. Systematic reviews have identified that in concussion, symptom recovery does not necessarily equate to physiological recovery [19]. For this reason, studies need to include outcomes beyond symptoms, as symptom improvement alone may not be a sufficient end-point for concussion management and it may not be a marker of neurophysiological recovery [19]. Our study will provide data that will help us understand whether there is a variable effect of exercise in children with concussion in comparison to their healthy peers. Such data will then help in understanding whether exercise, even if beneficial for symptoms, is eliciting a response following concussion that deviates from that observed in the control group. We will be able to examine whether there is an association between functional brain response to exercise and concussion symptoms (up to 28-days post-injury). Our study can also provide early and initial insight into whether a particular functional brain response to exercise is associated with the persistence of symptoms four weeks later.

Further, exercise-concussion research, supported by a large evidence base pointing to its efficacy and safety [38, 44, 47], is now taking aim at understanding how to optimize an “exercise prescription”. Studies are now beginning to examine how total volume of aerobic exercise impacts recovery, with early evidence suggesting that increasing exercise volume within the first 1–2 weeks of concussion can further improve symptom recovery [48, 49]. Other studies have reported that beginning exercise earlier post-concussion is more effective than exercise which commences in the later stages of recovery [50–52]. As additional exercise optimizations are made, it is important to ensure that more than one outcome is studied to understand exercise effects. More specifically, focusing on symptoms may not provide a holistic understanding of how exercise is impacting the physiology of concussion. Our study can provide insight into what areas of the brain are most responsive to exercise (with respect to changes in resting state brain activity), which other exercise intervention studies can then study as secondary outcomes. Alternatively, by improving our understanding of the cardiopulmonary response to exercise in concussion, our study can provide exercise-based targets for intervention as well. For example, if we observe that response to exercise is attenuated (as hypothesized) with respect to certain markers, such as minute ventilation, return-to-play decision making may consider not only an asymptomatic status but also a “normal” cardiorespiratory exercise profile as a marker of recovery as well. This may allow the field to optimize and personalize the exercise prescription for concussion not only in service of concussion symptoms, but also the underlying neuropathology of concussion itself.

To sum up, our study will provide mechanistic insight into how exercise impacts the brain following pediatric concussion. These insights may allow for the optimization of exercise protocols, or the development of protocols that are targeted at not only improving symptoms but also functional brain recovery. This mechanistic insight can help advance the exercise-concussion research frontier to drive more investigation into exercise effects not only at the level of symptoms, but also at the level of the brain.

### Electronic supplementary material

Below is the link to the electronic supplementary material.

Supplementary Material 1

## Data Availability

No datasets were generated or analysed during the current study.

## Abbreviations

fMRI: Functional magnetic resonance imaging
OUES: Oxygen uptake efficiency slope
RPE: Rating of perceived exertion
rsfMRI: Resting-state functional magnetic resonance imaging
TBI: Traumatic brain injury
V̇CO_2_: Maximal carbon dioxide production
V̇O_2_: Maximal oxygen uptake
